# Application of Video Laryngoscopy for Minimally Invasive Surfactant Therapy: A Retrospective Comparative Cohort Study

**DOI:** 10.3390/biomedicines12030618

**Published:** 2024-03-09

**Authors:** Tamara Jahmani, Michael R. Miller, Orlando da Silva, Soume Bhattacharya

**Affiliations:** 1 Department of Pediatrics, Western University, London, ON N6A 3K7, Canada; 2 Children’s Health Research Institute, 800 Commissioners Road East, London, ON N6C 2V5, Canada

**Keywords:** minimally invasive surfactant therapy (MIST), video laryngoscope, neonates, respiratory distress syndrome (RDS)

## Abstract

Minimally invasive surfactant therapy (MIST) has emerged as a preferred method of surfactant delivery. Pioneers of this technique have described the use of direct laryngoscopy (DL) for MIST. With the increasing application of video laryngoscopy (VL) for neonatal airway management, it is speculated that MIST techniques can be adapted for use with VL. Objective: To compare procedural success, operator ease of use, and complication of MIST using VL vs. MIST using DL. Methods: This was a retrospective, observational cohort study conducted at a tertiary-level neonatal intensive care unit after obtaining ethical approval. We included neonates who received MIST between 1 October 2020 and 31 October 2022. Baseline demographic characteristics, along with procedural data, were collected. Primary outcome measures included the overall procedural success rate, the need for multiple attempts, and the total number of attempts. Secondary outcome measures included the occurrence of adverse events, the need for a second dose of surfactant, and the need for intubation within 7 days of the procedure. Means and SDs, independent *t*-tests, frequencies, and chi-square were used as appropriate. *p*-values < 0.05 were considered statistically significant. Results: Of the 79 neonates included, 37 neonates received MIST via VL, while 42 received MIST via DL. The median gestational age was lower in the VL group at 29.0 weeks vs. 30.5 weeks (*p* = 0.011) in the DL group. The median birthweight in the VL group was 1260 g, IQR (1080, 1690), which was significantly lower than the DL group, which was 1575 g, IQR (1220, 2251), *p* = 0.028. Purpose-built catheter use was higher in the DL group. The overall procedural success was similar between groups. The need for multiple attempts was lower with VL in comparison to DL [4 (11%) vs. 13 (31%); *p* = 0.034)] at the univariate level but not significant at multivariate analysis (*p* = 0.131). Procedural complications, the need for a second dose of surfactant, the need for mechanical ventilation post-MIST, and operator ease of use were similar. User comments emphasized the value of VL in providing real-time visual information to confirm catheter placement and guide operators/trainees. Conclusion: Overall, in our cohort, despite VL being a more recently adapted technology used more in smaller, sicker, and more premature neonates, procedural success, complications, and operator ease of use for MIST using VL and DL were comparable. Our findings show the successful application of VL for MIST and suggest procedural advantages that might facilitate universal adoption.

## 1. Introduction

Intratracheal surfactant instillation remains the cornerstone of respiratory distress syndrome (RDS) treatment in premature neonates [1]. The intratracheal delivery of a surfactant can be accomplished by an endotracheal tube using a technique termed ‘intubate surfactant extubate (In-Sur-E)’ [2]. However, in the last decade, newer alternative techniques, termed minimally invasive surfactant therapy (MIST) or less-invasive surfactant administration (LISA), have emerged as preferred methods of surfactant delivery. MIST methods involve surfactant instillation via the intratracheal placement of a thin catheter in a spontaneously breathing neonate. This technique precludes the need for positive pressure ventilation, allowing surfactant delivery in stable neonates who are spontaneously breathing on non-invasive ventilation (NIV) [3,4,5,6]. Studies have shown premature neonates who receive surfactant via MIST have less need for intubation, a shorter duration of mechanical ventilation (MV), and a lower incidence of bronchopulmonary dysplasia (BPD) and severe intraventricular hemorrhage (IVH) [5,6,7].

MIST procedures that have been adopted in various countries often demonstrate practice variations, such as varying types of catheters, types of surfactants, choice of premedications, etc. [6,8,9]. The type of laryngoscope used to conduct MIST is now emerging to be another such procedural variation. The pioneers of MIST have described the use of direct laryngoscopy (DL) for minimally invasive surfactant delivery [10]. This is secondary to the fact that DL has been the traditional method for intubating neonates. The last two decades have witnessed the expanding clinical application of video laryngoscopy (VL). Technological and engineering advances have allowed it to be adapted for use, even in extremely premature neonates [11]. The use of VL has shown improvement in airway visualization and a reduction in the number of intubation attempts [12]. A recent systematic review that included eight studies encompassing 759 neonatal intubations showed that VL may increase the success of intubation on the first attempt and likely result in reduced incidence of airway-related adverse effects [11].

With the increasing application of VL for neonatal airway management, it is speculated that MIST techniques can be easily adapted for use with VL. In a Canadian survey, 47% of the neonatal intensive care units (NICUs) in Canada used VL for MIST procedures [8]. Recently, a quality improvement initiative showed the safety and efficacy of VL use in LISA [3]. MIST via VL could potentially offer procedural advantage, enhance procedural ease, and facilitate the universal adaptation of MIST methods. However, there is a paucity of data on this matter. The purpose of the present study was to compare procedural success, procedural ease, and complication of MIST using VL vs. MIST using DL.

## 2. Materials and Methods

### 2.1. Study Design

This was a retrospective cohort study conducted at a tertiary NICU in Southwestern Ontario, Canada. This regional Level 3 center has high inborn and outborn admission rates annually and provides a higher level of care to 18 Level 1 and Level 2 hospitals. This study was approved by the local institutional ethics board (HSREB No. 119981).

### 2.2. Eligibility Criteria

We included all neonates who received a surfactant via the MIST method as per our NICU guidelines from 1 October 2020 to 31 October 2022. We excluded neonates who received a surfactant by the In-Sur-E method, neonates suspected to have chromosomal or congenital anomalies, and newborns who received MIST without data regarding the type of laryngoscope used.

### 2.3. MIST Procedure Details

In our institute, the MIST procedure is routinely used for surfactant administration in spontaneously breathing neonates with a gestational age of ≥26 weeks gestation and RDS (FiO2 >/+ 0.30 on non-invasive respiratory support with a mean airway pressure (MAP) of ≥8 cm H20). Neonates with poor respiratory drive (defined as >3 apneic episodes in the hour preceding surfactant delivery), hypotension, birth asphyxia, and major congenital anomalies were not considered candidates for MIST. Premedication with fentanyl 0.5 mcg/kg intravenous (IV) and atropine 20 mcg/kg IV was used for procedural sedation and analgesia. Oral sucrose and containment measures were encouraged. Prior to the procedure, gastric contents were aspirated using a nasogastric/orogastric tube to minimize aspiration risk and to facilitate interpretation of the post-procedure gastric aspiration.

Trained physicians (subspecialty residents/staff neonatologists) and respiratory therapists performed the procedure. Neonates were positioned in a sniffing position and received nasal intermittent positive pressure ventilation (NIPPV) using nasal prongs for the entire procedure. DL was performed using a GREENLINE Fiberoptic Laryngoscope^®^, blade size 00 and up (Airlife, Grand Rapids, MI, USA; https://www.life-assist.com/products/details/879/greenline-fiber-optic-laryngoscopes/, accessed on 3 March 2024). The VL used during the study period was a C- MAC Video Laryngoscope Series 8403 Karl Storz ^®^, with blade sizes 0 and 1 Miller, Macintosh Curved #2 (Karl Storz SE & Co., Tuttlingen, Germany, https://www.karlstorz.com/us/en/category.htm?cat=1000104608, accessed on 3 March 2024).

Catheter insertion depth was calculated as 6 cm + weight in kilogram (kg) measured from the lip. Cords were visualized either using DL or through a VL followed by placing the catheter carefully through the vocal cords at the desired depth. After successful catheter placement, the laryngoscope blade was withdrawn. Then, Bovine Lipid Extract Surfactant (BLES^®^) at 5 mL/kg was administered slowly over 1 to 3 min. During the process, efforts were made to keep the neonate’s mouth closed to facilitate the continued delivery of positive airway pressure. Post-procedure, a gastric aspiration was completed to assess for accidental installation in the esophagus and/or substantial pharyngeal reflux. To perform this, a 10 mL syringe was attached to the end of a nasogastric/orogastric tube, and stomach contents were aspirated. The aspirate amounts were classified as none when no aspirate was obtained, minimal when <1 mL of aspirate volume was obtained, moderate when 1–5 mL of aspirate was obtained, and large when >5 mL of aspirate was obtained. As per the institutional protocol, there were a maximum of three attempts. Any introductions of laryngoscope blades followed by removal were defined as ‘attempts.’ Failed attempts refer to the failure to place the intratracheal catheter or the displacement of the placed catheter without completing surfactant delivery.

During the specified study period, a variety of catheters were used, including 16 G Angiocath, as well as purpose-built catheters, such as BlesCath™ (BLESCath, London, ON, Canada) and Surfcath™ (Vygon (UK) Ltd., Swindon, UK). A local quality improvement committee (QIC) oversaw the safe and effective implementation of MIST and used predesigned procedural forms (Appendix A) for the ongoing surveillance of procedural execution. The type of laryngoscope used was based on equipment availability and provider preference.

### 2.4. Data Collection

Baseline demographic characteristics, including gestational age, weight, antenatal steroids, mode of delivery, resuscitation details, and respiratory support before the procedure, were collected from patient charts. Data regarding the procedure were extracted from the completed procedural forms and included the type of catheter, type of operator, premeditations used, type of laryngoscope, number of attempts, successful delivery of surfactant, oxygen requirement post-surfactant, and the subsequent need for mechanical ventilation and/or need for a second dose of surfactant.

Data regarding complications, including apneas, desaturations (oxygen saturation < 85%), bradycardia (heart rate < 100 beats/min), the need to use positive pressure ventilation (PPV)/the need to intubate during the procedure, and air leak, were also collected. The operator’s perceived ease of use was collected from the procedural form record that collected the ease-of-use data on a 5-item scale ranging from ‘very easy’ to ‘very difficult regarding the laryngoscope and the MIST catheter.

### 2.5. Study Approach and Outcome Measures

All neonates who met the study eligibility criteria were divided into two groups based on the type of laryngoscope used (VL and DL). The baseline demographic data, procedural data, and complications were then compared between the two groups (VL vs. DL). Primary outcome measures included the overall procedural success rate. Procedural success was defined as the intratracheal instillation of a complete dose of surfactant with minimal to no surfactant aspirated on post-procedure gastric aspiration, the need for multiple attempts, and the total number of attempts. Secondary outcome measures included the occurrence of adverse events such as apnea, bradycardia, desaturation need for PPV, the need for a second dose of surfactant, the need for intubation within 7 days of the procedure, and procedural ease of use. Procedural ease of use was based on reports where users rated their experience as very easy, relatively easy, neither easy nor difficult, somewhat difficult, or very difficult.

### 2.6. Statistical Analysis

Continuous variables were summarized using median and interquartile ranges (IQRs), and group comparisons were conducted using Mann–Whitney U tests. Categorical variables were summarized using frequencies (%), and group comparisons were conducted using chi-square tests (or Fisher’s exact chi-square, when appropriate). Variables significant at the bivariate level were entered into a multivariable logistic regression model. *p*-values < 0.05 were considered statistically significant, and SPSS v.29 (IBM Corp., Armonk, NY, USA) was used to conduct all analyses.

## 3. Results

**Figure 1:**
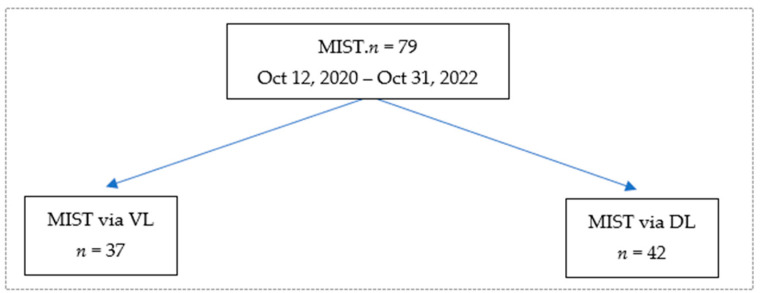
Study cohort. Abbreviations: MIST: minimally invasive surfactant therapy; VL: video laryngoscopy; DL: direct laryngoscopy.

A total of 79 neonates met the study eligibility criteria in the defined study period. A total of 37 neonates received MIST using VL, whereas 42 neonates received MIST via DL (Figure 1).

The baseline clinical and demographic characteristics in the two groups are summarized in Table 1. The median gestational age was lower in the VL group at 29.0 weeks vs. 30.5 weeks in the DL group, *p* = 0.011. The median birthweight in the VL group was 1260 g, IQR (1080, 1690), which was significantly lower than the DL group, which was 1575 g, IQR (1220, 2251), and *p* = 0.028. The distribution of catheter types between the two groups was different, with a higher use of the purpose-built catheters in the DL group. The mode of respiratory support prior to MIST was similar in the groups; however, the peak inspiratory pressure (PIP) before the procedure was higher in the VL group than in the DL group, but the difference was not significant (17 vs. 16 cm of H_2_O, *p* = 0.878).

### 3.1. Procedural Outcomes

The procedural success and complications in the two groups are summarized in Table 2. The overall procedural success was similar between the two groups. In the VL group, 100% procedural success was noted, and in the DL group, success was 95.2%. The need for multiple attempts was lower in the VL group, with only four (11%) patients needing multiple attempts vs. thirteen (31%) needing multiple attempts in the DL group (*p* = 0.034). Procedural complications (apnea, bradycardia, need for PPV, need for intubation during the procedure, and air leaks) were similar in the two groups. The need for a second dose of surfactant and the need for mechanical ventilation within 7 days of MIST were similar in the two groups. Incorporating the baseline variables that were significantly different in the two groups (GA, birthweight, PEEP prior to MIST, and type of catheter), the difference in the need for multiple attempts was no longer significant (*p* = 0.131) in the multivariable regression analysis.

### 3.2. Operator Ease of Use Analysis

**Figure 2:**
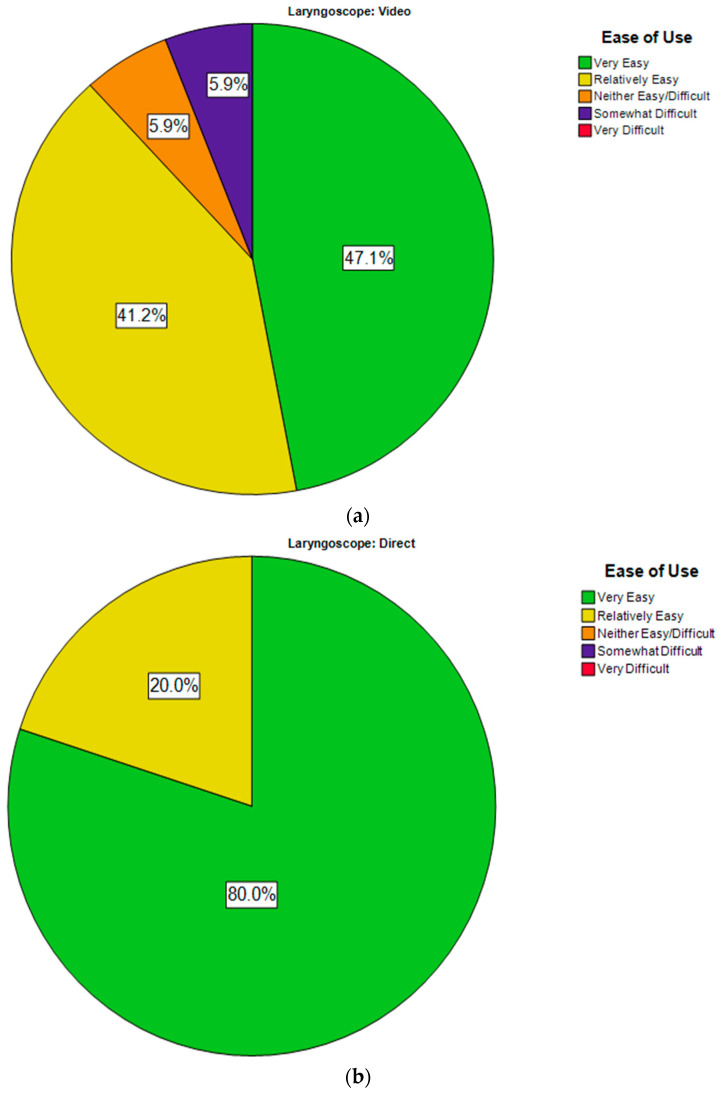
(**a**) Operator ease-of-use VL. (**b**) Operator ease-of-use DL.

The subjective ease of use reported by the operators was similar in the two groups; all DL operators found the procedure relatively easy to very easy (80%), whereas 89% of VL operators found the procedure relatively easy to very easy (Figure 2a,b). None of the users found the procedure very difficult with either laryngoscope. Only two VL users (5.9%) reported the VL to be ‘somewhat difficult’.

## 4. Discussion

Our study compared procedural success, the need for multiple attempts, procedural complications, and operator ease of use among MIST using VL vs. DL. Data from our cohort showed that the need for multiple attempts was significantly lower in the VL group at the bivariate level. In our cohort, VL was used in more premature neonates who weighed less and who needed greater respiratory support. Despite the high-risk features in the VL group, procedural success, attempts, and complications were similar in the two groups.

Our study shows that the use of VL for MIST is safe, effective, and comparable to DL for MIST. Our results suggest that VL may provide a slight procedural advantage in terms of the number of attempts. This finding is interesting because the VL was used more frequently in the cohort with smaller birth weights and younger age groups, which is typically a more challenging subset of neonates to intubate. Additionally, custom-built catheters were more frequently used with DL. Despite not having the advantage of custom-built catheters, the VL group had similar success and complication rates. The procedural advantage of VL has been seen in pediatric airway management. A recently published large multi-center randomized controlled study reported that in neonates and infants, the first-attempt tracheal intubation success rate with no desaturation was higher with VL (89·3% [95% CI 83·7 to 94·8]; *n* = 108/121) compared with DL (78·9% [71·6 to 86·1]; *n* = 97/123) [12]. In 2023, Tippman et al. reported the findings of their randomized controlled trial and showed that first-attempt nasotracheal intubation in neonates was higher with VL and, notably, there was no esophageal intubation with VL [13]. Lingappan et al. conducted a metanalysis in 2023 that showed that VL increased the chance of first-attempt intubation and resulted in fewer intubation attempts but did not reduce the time required for completing successful intubation. The results of this metanalysis also suggested that the VL likely decreased airway-related adverse effects [11]. While these studies have demonstrated the safe use of VL for endotracheal intubation, our study is the first to compare procedural success with VL vs. DL in the context of MIST. Although our study did not demonstrate an increase in the procedural success rate with VL, our rates were high and comparable to MIST with DL. A larger sample size and head-to-head comparison may demonstrate a procedural benefit. The trend towards fewer attempts in VL-assisted MIST keeps with the findings reported in the literature about VL and neonatal intubation. The net procedural time is another outcome of interest in the context of MIST. It is a major imperative to minimize the procedural time for MIST to avoid complications, such as apnea or significant bradycardia. Our study did not investigate the total time taken to complete the procedure in VL MIST vs. DL MIST, but future studies exploring this would be interesting.

In our study, the procedural complications were explored in both groups, and desaturations remained the most common complication seen. Bradycardia was not seen frequently, which is possibly related to the premedication with atropine. Post-procedure gastric aspirate volumes were checked for accidental instillation in the esophagus. Some amount of aspirate is expected normally, as pharyngeal reflux is common with MIST. However substantial aspirate volume may suggest that the catheter was not in an optimal position. Our results did not demonstrate any significant difference in the gastric aspirate volume between the two groups.

In MIST, accurate endotracheal placement of the thin catheter is dependent on operator experience, as there is no objective method to confirm endotracheal placement. During endotracheal intubation, various methods can be used to confirm appropriate placement, such as colorimetric carbon dioxide measurement, end titer CO_2_ measurement, capnography, or X-ray [14]; none of these methods can be used in the context of MIST. VL use for MIST provides an advantage in this regard, allowing superior airway visualization and visual confirmation of appropriate placement by not only operators themselves but allowing supervisors/assistants to participate in the real-time visual confirmation of adequate placement and positioning of the MIST catheter.

For this study, we evaluated operator experience with regard to the laryngoscope used. This was initiated by the local QIC group using the predesigned procedural form (Appendix A). The operators documented their user experiences in real time, and the research team then retrospectively analyzed the operator’s experience. This was particularly relevant, as the VL was a relatively new addition to the NICU, and most of the operators had greater experience with traditional DL. Based on the operator ease of use, it appeared that operators using VL found it very easy to relatively easy, and the overall subjective ease of use in the two groups was comparable. User comments reflected a common theme that VL had the benefit of having multi-person airway visualization.

Additionally, the use of VL can expand opportunities for education and training. Traditionally, MIST has been restricted to experienced incubators. With the advantage of real-time visualization and guidance, trainees or operators with limited experience can be better facilitated in performing a successful MIST procedure. In turn, this might improve the educational experience of pediatric trainees. This is particularly relevant as there is considerable concern that opportunities for trainees to gain skills in neonatal airway management are limited [15]. VL also provides the opportunity to record and archive procedural images that can complement clinical documentation. All supervisors agreed on the inherent educational advantage that allowed real-time visual information-based feedback that improved both the educator and trainee experience. This is in keeping with the theme that has been published in the literature exploring the applications and advantages of VL [16].

VL may also allow broader application and adaptation of MIST among Level 2 neonatal centers that are still using traditional methods of surfactant delivery, such as In-Su-Re. Future considerations, such as using technology to provide real-time remote assistance to MIST in Level 2 neonatal centers, are exciting prospects.

We acknowledge that our study is limited by its retrospective nature and its relatively small sample size. The use of a laryngoscope was determined by equipment availability and operator preference. Operator preference often stems from their previous training and experience, and hence is a source of potential selection bias. Multiple catheter types were used during the study period, which also could be a potential source of bias. However, our study is the first study to present pragmatic real-world data on different types of laryngoscopes and their impact on the MIST procedure. Our study is also unique in its attempt to capture the operator’s experience. Our data about the high procedural success rate with MIST with VL should encourage other institutions to adopt VL-guided MIST. Future prospective studies that explore the success rates of VL with operators at different levels of experience, trainee experience and opportunity, total procedural time with VL MIST, and the application of VL-guided MIST in Level 2 centers using remote technology guidance would be interesting.

## 5. Conclusions

Our study compared procedural success, the need for multiple attempts, procedural complications, and operator ease of use among MIST using VL vs. DL. Procedural success, complications, and operator ease of use for MIST using VL and DL were comparable, despite VL being a more recently adapted technology and being used more in smaller, sicker, and more premature neonates. Our findings show the successful application of VL for MIST and suggest a procedural advantage that might facilitate universal adoption.

## Figures and Tables

**Table 1:** Baseline clinical and demographic characteristics in the cohort.

	Video Laryngoscopy (*n* = 37)	Direct Laryngoscopy (*n* = 42)	*p*-Value
Gestational age in weeks, median (IQR)	29.0 (28, 30)	30.5 (29, 34)	0.011
Sex, *n* (%)			0.080
Male	24 (65)	19 (45)	
Female	13 (35)	23 (55)	
Mode of delivery *n* (%)			0.932
SVD	12 (32)	14 (33)	
C/S	25 (68)	28 (67)	
Birth weight in grams, median (IQR)	1260.0 (1080, 1690)	1575.0 (1220, 2251)	0.028
APGAR score at 5th min, median (IQR)	8.0 (7, 9)	8.0 (6, 9)	0.342
APGAR Score at 10 min, median (IQR)	9.0 (7, 9)	8.0 (8, 9)	0.616
Antenatal steroid, *n* (%)			0.187
None	6 (16.2)	13 (31)	
Any (complete and incomplete)	31 (83.2)	29 (69)	
Professional performing MIST, *n* (%)			0.818
RT	21 (58.3)	27 (64.3)	
Fellow	14 (38.9)	14 (33.3)	
MD	1 (2.8)	1 (2.4)	
Type of catheter used, *n* (%)			0.006
BLEScath™	8 (21.6)	12 (32.4)	
SurfCath™	16 (43.2)	23 (62.2)	
Angiocath	13 (35.1)	2 (5.4)	
Postnatal age at MIST in hours, median (IQR)	5.8 (2, 14)	5.2 (3, 12)	0.626
FiO2 before the procedure, median (IQR)	36.0 (30, 40)	35 (30, 45)	0.652
Type of NIV before MIST, *n* (%)			0.092
CPAP	4 (11.1)	11 (26.2)	
NIPPV	32 (88.9)	31 (73.8)	
PEEP prior to MIST,			0.025
median (IQR)	8.0 (7, 8)	8.0 (7, 8)	
Mean (SD)	7.39 (0.89)	8.3 (2.23)	
PIP before MIST, median (IQR)	17.0 (15,18)	16.0 (16, 18)	0.878
Use of premedication, *n* (%)			
Atropine	34 (94.4)	41 (97.6)	0.593
Fentanyl	34 (94.4)	42 (100.0)	0.210
Sucrose	23 (65.7)	22 (52.4)	0.237

Abbreviations: M: mean, SD: Standard Deviation, *n*: number of neonates, MIST: minimally invasive surfactant therapy, MD: medical doctor, RT: respiratory therapy, FiO2: fraction of inspired oxygen, NIV: non-invasive ventilation, CPAP: continuous positive airway pressure, NIPPV: non-invasive positive pressure ventilation, PEEP: positive end-expiratory pressure, PIP: peak inspiratory pressure, IQR: interquartile range.

**Table 2:** Comparison of procedural success and procedural complications in the two groups.

	Video Laryngoscopy (*n* = 36) *	Direct Laryngoscopy (*n* = 42)	*p*-Value
Overall Procedural Success *n* (%)	36 (100)	40 (95.2)	0.497
Need for Multiple Attempts *n* (%)	4 (11.1)	13 (31)	0.034
Number of Attempts M, (SD)	1.3 (0.8)	1.5 (0.8)	0.53
Complications, *n* (%)			
Apnea	16 (44)	19 (45)	0.944
Bradycardia	1 (2.8)	0 (0)	0.462
Desaturation < 85%	24 (66.7)	30 (71.4)	0.650
Need for PPV	2 (5.6)	1 (2.4)	0.593
Need to Intubate during	0%	0%	
Procedure			
Air Leaks	(3) 8.1%	(5) 12.2%	0.751
Amount of Surfactant			0.129
Aspirated from the Stomach, *n* (%)			
None	17 (89.5)	23 (60.5)	
Minimal	2 (10.5)	11 (28.9)	
Moderate	0 (0)	3 (7.9%)	
Large	0 (0)	1 (2.6)	
Need for a Second Dose of Surfactant *n* (%)	4 (10.8)	7 (16.7)	0.453
Need for Intubation within 7 days of Procedure *n* (%)	4 (10.8)	8 (19.0)	0.309

Table 2 abbreviations: M: mean, SD: Standard Deviation, *n*: number of neonates, PPV; positive pressure ventilation; * missing data in 1 patient.

## Data Availability

Available upon request from the corresponding author.

## Appendix A

Date of MIST Delivery	Time of MIST Delivery:	Dose of Surfactant O. First O. Second	
Ventilation support when MIST decided	○ CPAP ○ PEEP______ ○ FIO2______	○ NIPPV ○ PEEP______ PIP________ ○ FIO2_______	
**MIST PROCEDURE**			
Atropine	○ Yes	○ No ○ If No Why: _______________	
Fentanyl Given	○ Yes ○ Dose:_____________ ○ Number of doses:	○ No ○ If No Why: _______________	
Sucrose	○ yes	○ No	
Bradycardia (<100 bpm)	○ Yes	○ No	
Apnea	○ Yes	○ No	
Desaturation (SpO_2_ < 85%)	○ Yes	○ No	
Need for PPV during procedure	○ yes	○ No	
Need to Intubate during procedure	○ yes	○ No	
Amount of Surfactant Obtained on Gastric Aspiration	○ None ○ Minimal ○ Moderate to Large Volume ○ Not Done		
Able to deliver full dose of surfactant	○ Yes	○ NO ○ If no explain:	
Multiple Attempts Needed To deliver Surfactant By MIST	○ Yes ○ Number of Attempts-__	○ No	
**PROCEDURE PREFORMER**			
	Attempt 1	Attempt 2	Attempt 3
Profession:	RT- Fellow, Resident, NP, Staff	RT- Fellow, Resident, NP, Staff	RT- Fellow, Resident, NP, Staff
Laryngoscope:	O. Direct O. Video	O. Direct O. Video	O. Direct O. Video
Type of Catheter:			
Ease of use (Laryngoscope)	○ Very easy ○ Relatively Easy ○ Neither Easy nor Difficult ○ Somewhat difficult ○ Very Difficult	○ Very easy ○ Relatively Easy ○ Neither Easy nor Difficult ○ Somewhat difficult ○ Very Difficult	○ Very easy ○ Relatively Easy ○ Neither Easy nor Difficult ○ Somewhat difficult ○ Very Difficult
Ease of Use of Catheter	○ Very easy ○ Relatively Easy ○ Neither Easy nor Difficult ○ Somewhat difficult ○ Very Difficult	○ Very easy ○ Relatively Easy ○ Neither Easy nor Difficult ○ Somewhat difficult ○ Very Difficult	○ Very easy ○ Relatively Easy ○ Neither Easy nor Difficult ○ Somewhat difficult ○ Very Difficult
Additional Comments or Complications:			
